# Platypnea-Orthodeoxia Syndrome: A Rare and Treatable Cause of Positional Dyspnea

**DOI:** 10.7759/cureus.9052

**Published:** 2020-07-07

**Authors:** Karan Puri, Ghulam Aftab, Arjun Madhavan, Keval V Patel, Megha Puri

**Affiliations:** 1 Internal Medicine, Saint Peter's University Hospital, New Brunswick, USA; 2 Pulmonary Medicine, Saint Peter's University Hospital, New Brunswick, USA; 3 Critical Care, Saint Peter’s University Hospital, New Brunswick, USA; 4 Cardiology, Saint Peter’s University Hospital, New Brunswick, USA; 5 Cardiology, Rutgers-Robert Wood Johnson University Hospital, New Brunswick, USA; 6 Pathology, Pandit Bhagwat Dayal Sharma University of Health Sciences, Rohtak, IND

**Keywords:** orthodeoxia, platypnea, intracardiac shunts, intrapulmonary shunts, hyperoxia test

## Abstract

Platypnea-orthodeoxia means low oxygen saturation and dyspnea in the upright posture which improves on lying down. The causes can be classified into the intrapulmonary shunt, intracardiac shunt, and ventilation-perfusion mismatch.

A 62-year-old male presented with shortness of breath, which had worsened over a period of one year. Various investigations were done to rule bacterial, viral infection, pulmonary embolism, and other respiratory and cardiac causes. The initial echocardiogram showed an ejection fraction of 55%. The patient was observed to be having dyspnea only in the upright position. In the recumbent position, the dyspnea disappeared with a marked improvement in oxygen saturation. A repeat echocardiogram with a bubble study was done which showed an atrial septal defect. Surgical closure of the defect was performed which improved the patient’s oxygen saturation to baseline normal. This case demonstrates that a vigilant approach is required in cases of dyspnea, keeping in mind the not-so-common phenomenon like platypnea-orthodeoxia syndrome

## Introduction

Orthodeoxia means low oxygen saturation in the upright posture and improvement when lying down. Platypnea is dyspnea in an upright position, which improves with recumbency [1]. It is an uncommon and often overlooked cause of dyspnea. Causes can be classified into an intracardiac shunt, intrapulmonary shunt, and ventilation-perfusion mismatch [2].

## Case presentation

A 62-year-old male presented with a history of shortness of breath for one year, with his condition worsening over the past two to three months. Initially, he was able to walk three to four miles a day but has been able to walk only half to one mile over the last year. He gave a remote two-year history of cigarette smoking half a pack per day about 25 - 30 years ago. He was admitted to the hospital with similar complaints three months prior to his presentation. A computed tomography angiogram (CTA) of the chest done at that time showed no pulmonary embolism and no consolidation/parenchymal changes. An echocardiogram demonstrated an ejection fraction of 55% with a normal right ventricle and no valvular abnormality. The viral panel was positive for rhinovirus and he was deemed to have dyspnea because of the rhinovirus infection. On discharge, he was comfortable on room air with no active complaints, although he felt he was not back to his baseline exercise capacity.

Sometime later, the patient went to his primary care physician for persistent shortness of breath. To rule out coronary ischemia, a left heart catheterization was performed which showed normal coronary arteries.

The patient then presented to the pulmonary clinic with hypoxemia. Even with supplemental oxygen, the hypoxemia persisted. However, when asked to lay flat, his oxygen saturation improved to 95%. The patient’s previous CTA was reviewed and no abnormalities were seen. He was advised to go to the hospital emergency room (ER) for admission.

The patient was initially placed on a 3 liters (L)/minute nasal cannula without improvement in oxygen saturation. He was subsequently placed on a non-rebreather mask and his saturation improved to 98%.

A complete blood count and comprehensive metabolic panel were normal. Arterial blood gas analysis performed on the non-rebreather mask showed a high alveolar-arterial gradient. A CTA was repeated to evaluate a pulmonary cause of platypnea-orthodeoxia syndrome. No pulmonary abnormality was detected.

**Figure 1:**
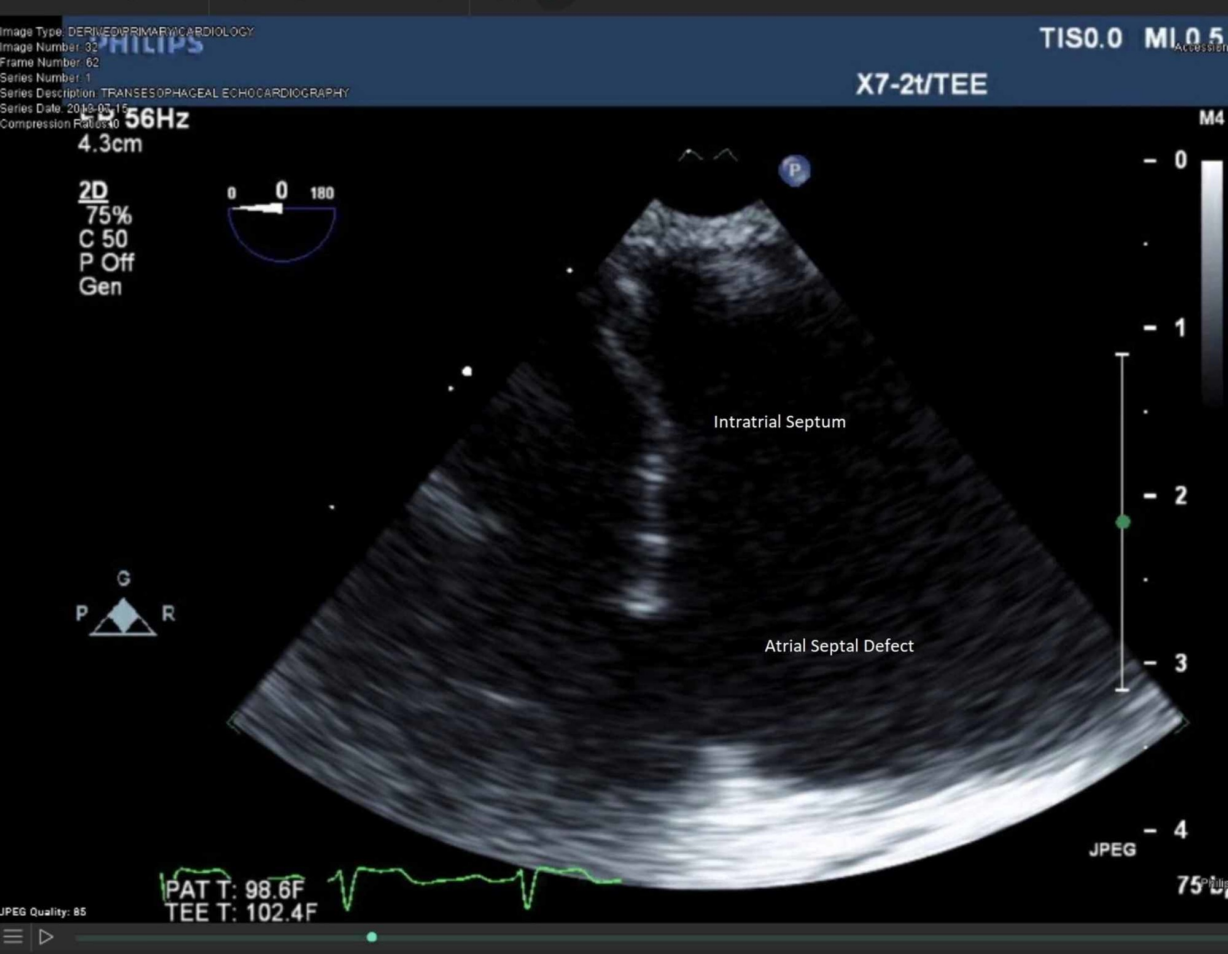
Transesophageal echocardiogram showing an atrial septal defect

An echocardiogram was performed which showed an atrial septal defect (ASD) on a bubble study and was confirmed on a transesophageal echocardiogram with right-to-left shunting (Figure 1). The patient was transferred to another facility, where the closure of the ASD was done. On follow-up appointments, the orthodeoxia and platypnea had both resolved.

## Discussion

Platypnea-orthodeoxia syndrome is caused mainly due to intrapulmonary shunts like pulmonary arteriovenous malformations and hepatopulmonary syndrome. Rare cardiac causes include patent foramen ovale and atrial septal defect (ASD). Burchell was the first to describe a case of platypnea orthodeoxia syndrome over half a century ago [3].

Usually, interatrial communication may cause a shunt from the left to the right side of the heart as the left atrium has higher pressures than the right atrium and the right ventricle has greater compliance compared to the left [4]. A right to left interatrial shunt may be associated with pulmonary hypertension; however, this is rare without a right to the left pressure gradient.

The rarity of cardiac shunts causing this syndrome is due to the high left atrial pressure which causes the foramen ovale to close. However, it can occur in a right to left shunt or due to anatomical distortion where the inferior vena cava (IVC) comes in line with the ASD, even without an elevated right to the left pressure gradient. Thus, the blood flows from the right side of the heart to the left and from IVC to ASD, respectively [2, 5-6].

Diagnosis is made when hypoxemia and dyspnea are related to the upright posture and improve on lying down. For our patient, the hypoxemia and dyspnea both improved on lying down. The hyperoxia test is performed wherein 100% oxygen is applied and the patient's partial pressure of oxygen (PaO_2_) is measured in the supine and upright positions [1]. As mentioned earlier, our patient’s oxygen saturation did not improve upon performing a hyperoxia test. An echocardiogram with a bubble study is done; if bubbles are seen after three cardiac cycles, it is likely to be an intrapulmonary cause; if seen within three cycles, it is likely to be a cardiac cause [2, 6]. In our patient’s case, bubbles were seen on the left side of the heart immediately - this showed the morphology of the platypnea-orthodeoxia syndrome was due to a cardiac cause. The gold standard for the diagnosis of platypnea-orthodeoxia syndrome is cardiac catheterization, which shows a drop in the oxygen saturation between the pulmonary vein and the aorta [1].

Treatment involves closure of the underlying defect. For ASD, either a transcatheter closure or open surgery is performed [7]. In our case, our patient had surgical closure of the ASD.

## Conclusions

Platypnea-orthodeoxia, being a rare cause of dyspnea, is likely to be underestimated. Therefore, an awareness of this entity is required to prevent any delay in the appropriate management of the patients.
